# Supplementary material for: Are carbon emissions trading and green financial instruments synergistic? -Comprehensive quantitative research based on content analysis
Source: PLoS One. 2024 Mar 7;19(3):e0298601. doi: 10.1371/journal.pone.0298601 (PMC10919601; doi:10.1371/journal.pone.0298601)
Supplement: S1 Data — (ZIP) [file pone.0298601.s001.zip › Data/地级市Z_F工作报告.zip!/║■▒▒/╛ú├┼╩╨/└┤╫╘╣1⁄2╓┌║┼í╛┬φ┐╦╩2╛▌═°í┐_2017.docx]

各位代表：

　　现在，我代表市人民政府向大会作工作报告，请予审议，并请市政协各位委员和其他列席人员提出意见。

　　一、过去五年工作回顾

　　过去的五年，是我市经济社会快速发展、人民群众得到实惠最多的五年。五年来，在省委、省政府和市委的坚强领导下，在市人大、市政协的监督支持下，我们紧紧团结和依靠全市人民，积极应对经济形势复杂多变、区域竞争日趋激烈、调整转型任务繁重、极端天气灾害多发等严峻考验，励精图治，奋力拼搏，谱写了荆门科学发展、跨越式发展的新篇章。

　　过去五年，是我市战略地位明显提升的五年。我们坚持观大势、谋大事、创大业，中国农谷建设、柴湖振兴发展上升为省级战略，中国农谷写入《长江经济带发展规划纲要》；确立了建设江汉平原中心城市、湖北新兴增长极、全国新型城镇化和城乡一体化示范区的战略定位，明确了生态立市、产业强市、资本兴市、创新活市的发展路径。我市成功创建国家园林城市、国家森林城市，被确定为国家节能减排财政政策综合示范市、国家循环经济示范市、国家现代农业示范区、国家农业科技园区、国家新型城镇化综合试点地区、全国节水型社会建设示范区。在2016年全国城市信用综合排名中，荆门位居全省首位。

　　过去五年，是我市综合实力明显增强的五年。全市主要经济指标增速高于全国、全省平均水平，地区生产总值迈上1500亿元新台阶，是2011年的1.6倍；固定资产投资、社会消费品零售总额、地方一般公共预算收入分别年均增长22.3%、14.2%、18.4%，总量分别是2011年的2.7倍、2.1倍、2.3倍。经济结构持续优化，三次产业结构比由2011年的17.7：52.4：29.9调整为14.0：51.9：34.1。农产品加工业产值与农业总产值之比由2.07：1提高到3.64：1，京山、沙洋、钟祥连续多年被评为全省“三农”发展成绩突出十强县（市）。工业经济总量稳居全省第4位，农产品加工成为“千亿产业”，规模以上工业企业从709家增加到1196家。现代服务业占地区生产总值比重提高2.7个百分点，限上商贸企业从266家增加到679家。荆门高新区跻身国家高新区行列，全市高新技术企业由23家增加到140家。县域经济实力持续增强，钟祥、京山、东宝稳居全省县域经济发展“第一方阵”。

　　过去五年，是我市城乡面貌明显改观的五年。全市常住人口城镇化率达到55.56%，比2011年提高7.4个百分点。中心城区建成区面积拓展到62平方公里，“三环八射”路网基本成型；漳河新区骨架逐步拉开，成为全省首批绿色生态示范城区。实施“五城同创”，关停葛洲坝水泥厂城区生产线，拆除海慧沟、关坡等片区56.5万平方米存量违建，数字化城市管理项目被授予“2015年中国人居环境范例奖”。钟祥摘得世界长寿之乡桂冠，京山成功创建全省首个国家生态县，沙洋成为江汉运河生态文化旅游带重要节点城市。10个镇跻身全国重点镇行列，66个村被命名为省级宜居村庄，151个村荣获省级绿色示范村称号。建成全国首个土壤修复产业园，漳河、惠亭湖、莫愁湖先后通过国家湿地公园验收，全市森林覆盖率达到32.4%。

　　过去五年，是我市改革步伐明显加快的五年。先后承担46项国家、省改革试点项目，沙洋土地按户连片耕种做法写入2016年中央1号文件，京山整合公共检测检验机构、钟祥彭墩一二三产业融合发展、全市医疗保险门诊统筹等经验在全国、全省推广，钟祥被确定为全国农村产业融合发展试点示范县（市）、农村承包土地经营权抵押贷款试点，京山被列为全省唯一的国家农村集体资产股份权能改革试点。市级行政审批事项减少60%，成立全国首个创新创业服务局，建成国家级小型微型企业创业创新示范基地1家、科技企业孵化器2家、“众创空间”1家。新增主板上市企业2家、“新三板”挂牌企业3家；四板挂牌企业达到258家，居全省第3位。累计引进市外境内资金4287.7亿元、实际利用外资12.9亿美元，分别年均增长32.8%、12.1%。海关荆门办事处恢复运行，荆门出入境检验检疫办事处（筹）正式开检，中兴农谷公用型保税仓库和全省首家县级保税仓库——京山恒泰源建成运营。

　　过去五年，是我市群众福祉明显增进的五年。扎实打好脱贫攻坚战，实施整村推进161个、贫困乡镇连片开发5个，全市24.7万人实现脱贫。城镇、农村常住居民人均可支配收入分别居全省第4位、第2位。累计新增城镇就业26.4万人，我市被评为全国创业先进城市；五项社会保险参保达198万人次；城乡低保、农村五保实现应保尽保；累计新建保障性住房、棚户区改造安置住房78152套，改造农村危房35682套，高标准完成南水北调中线工程移民安置任务，累计解决61.6万农村居民饮水安全问题。我市连续多年被评为国家科技进步先进市，义务教育均衡发展通过国家评估认定，职业教育服务地方发展能力进一步提升，“农家乐杯”荣获全国首届群众文化活动品牌，农村广播电视“村村响”“户户通”实现全覆盖，乡村农民体育健身工程覆盖率达到93.5%。成功创建3家三甲医院，全面推开县级公立医院改革，人口计生工作连续三年获得全省党政领导线考核一等奖。我市荣获全国法制宣传教育先进城市称号。“平安荆门”建设深入推进，安全生产和食品药品监管持续加强，信访维稳工作扎实有效，人民群众安全感和满意率持续提升。

　　国防动员和国防后备力量建设稳步推进，优抚安置、双拥工作成效显著。人防建设、应急管理、防灾减灾、气象服务不断加强，妇女儿童、老年人和残疾人权益得到有效保障，民族宗教、外事侨务、对外援建等工作取得新成绩，统计、机关事务、住房公积金、物价、档案、地方志、保密、新闻出版等工作迈出新步伐。

　　在加快经济社会发展的同时，我们不断加强政府自身建设，建立市政府常务会议学法制度，出台重大行政决策程序规定、政府合同管理等办法，积极参与地方立法工作，行政负责人出庭应诉率达到100%；自觉接受各方面监督，高质量办理人大议案、代表建议和政协提案；扎实开展党的群众路线教育实践活动、“三严三实”专题教育和“两学一做”学习教育，认真落实政府党组全面从严治党主体责任，强化财政监督、审计监督和行政监察，严厉查处各类违规违纪违法行为，政府执行力、服务力、公信力持续提升。

　　刚刚过去的2016年，是我市发展史上不同寻常的一年。我们牢牢把握新常态下发展的主动权，切实打好扩大投资关键之战、城市建设攻坚之战、抗灾救灾决胜之战，实现了“十三五”良好开局。全年实现地区生产总值1521亿元，增长8.5%，增幅居全省第3位；规模以上工业增加值增长9.7%；固定资产投资1531.46亿元，增长18.9%，增幅居全省第1位；社会消费品零售总额614.23亿元，增长13.4%；实际利用外资37630万美元，增长10.7%；出口总额97800万美元，增长11%；地方一般公共预算收入91.7亿元，增长14.1%，增幅居全省第3位；城镇常住居民人均可支配收入28920元，增长8.19%；农村常住居民人均可支配收入15811元，增长7.44%；居民消费价格涨幅2%；城镇登记失业率3.8%；人口自然增长率4.65‰。

　　一年来，我们主要抓了六个方面的工作：

　　（一）着力抓投资、稳增长，发展后劲切实增强。深入实施产业链招商行动计划，举办首届荆门楚商大会，实行每季度项目拉练制，长丰猎豹汽车、金泉新材料动力电池、上海拖拉机、大连数控机床、湖南泰富重装等79个投资10亿元以上的重大产业项目落户，436个亿元以上项目开工建设，260个亿元以上项目建成投产。狠抓重大交通基础设施项目建设，沙洋港建成开港，石牌港区综合码头、漳河机场改扩建工程竣工，蒙华铁路、枣潜高速公路、荆门城区绕城公路建设加快推进。制定促进民间投资健康发展政策，共策划重资产项目62个，建成160.84万平方米厂房；实施竹皮河流域水环境综合治理等24个PPP项目，落地项目数居全省第1位。

　　（二）着力抓产业、调结构，实体经济稳中向好。出台《关于进一步减轻税费降低工业企业成本的意见》，扎实开展“营改增”、降低融资担保费率等惠企改革，共为企业降低成本17.8亿元。开展政银企对接，全社会融资额368.7亿元，增长50%。新能源汽车、通用航空等战略性新兴产业加快发展，高新技术产业增加值占地区生产总值的比重达到12.8%。完成工业技改投资240亿元，同比增长30%。金鹰能源、广源食品等21家企业实现兼并重组，全市新增规模以上工业企业115家。加快发展现代服务业，万达广场等一批重大商贸项目建成运营，金瑞物流产业园成为首批全国智慧物流配送示范单位，与阿里巴巴集团等合作建设农村电子商务服务平台8个、服务站（点）1100个。全年实现旅游综合收入148亿元，增长14.6%。

　　（三）着力抓“三农”、促增收，现代农业加快发展。围绕“一谷双核、多点支撑”布局，加快建设中国农谷“三区三中心”，中国农谷院士村投入运营，甘薯、马铃薯、水生蔬菜等6个种业基地基本建成，彭墩长寿食品产业园开工建设，南方黑芝麻集团富硒食品全产业链项目落户。加快推动柴湖振兴发展，移民新城一期竣工，建成全国最大的盆花生产基地。大力发展生态高效农业，“一高三新”推广面积突破220万亩，新建标准化畜禽养殖示范场142个、现代渔业示范园区5个、林业特色产业基地8个。全市新型农业经营主体达到2.2万个，农业规模经营比重达到42.8%。实施土地整治33万亩，改善农田灌溉面积20万亩，主要农作物耕种收综合机械化水平达到77%。

　　各位代表，2016年，我市连续遭遇了六轮强降雨袭击，受灾面积之广、受灾人数之多、因灾损失之大历史罕见，我们众志成城、万众一心，奋力抢险救灾，全市未倒一堤、未溃一坝、没有发生一起疫情。及时组织抢种、补种、改种，实现了大灾之年农业增效、农民增收。市级筹资8亿元支持灾后恢复重建，并策划重大项目660个，总投资749.7亿元，一批水毁设施迅速修复，一批重大水利工程迅速启动实施，一批因灾受损和倒塌的房屋迅速修缮或重建，1825户群众可全部迁入新居！

　　（四）着力抓建设、强管理，城市品位不断提升。启动《2049荆门城市远景发展战略规划》编制，实施城建项目350个，完成投资186亿元，我市荣获“2016中欧绿色和智慧城市奖”产业推广奖。漳河新区建设全面提速，荆门绿色生态科技产业城核心岛项目启动实施，爱飞客航空小镇机场核心区基本建成，漳河镇入选全国首批特色小镇。老城区更新步伐加快，高标准推进象山大道及城区三条高速公路出入口沿线综合整治，对22家农贸市场、169个“三无”小区、614条背街小巷进行整治，创建国家卫生城市工作取得阶段性成效。完成荆钟、荆京、荆沙三条新型城镇化示范带总体规划编制，共实施支撑性项目85个。京山温泉新区、沙洋滨江新区、钟祥南湖新区建设加快推进。出台《关于坚持生态立市建设生态荆门的决定》，建成荆门植物园等一批精品绿化工程，全部关停中心城区10蒸吨以下燃煤锅炉，淘汰6590辆黄标车，PM10、PM2.5浓度分别同比下降13.2%、18.3%。扎实开展美丽宜居乡村建设，实施农村环境治理“八大行动计划”，推进52个乡镇污水处理厂建设，建成480处农村生活污水处理设施。

　　（五）着力抓改革、重创新，内生动力加速释放。认真落实“四个十”改革项目清单，切实疏通“堵点”、消除“痛点”。在全省率先编制市级政府部门权责清单、监管清单，市级行政审批事项减少到144项，行政审批再提速29.6%。实行“五证合一、一照一码”和“两证整合”，开展全国商务综合执法体制改革试点工作，净增市场主体12734户，企业注册资金增长62.98%。全市不动产登记工作整合到位、运转有序。开展税收共治，加强投融资监管和财政专项资金清单制管理，进一步完善市区财政管理体制。推进公共资源交易平台整合，交易规模突破200亿元。实施农业大部制改革，完成农村土地承包经营权确权登记颁证工作，市农村产权交易中心建成运行。实施园办一体化改革，荆门高新区与掇刀区实现一体化发展。深化国有企业改革，重组成立农谷投资有限公司、城市建设投资控股集团、交通建设投资有限公司。深化投融资体制改革，设立总规模180亿元的特色产业基金和城市发展基金，启动农产品加工、再生资源利用与环保等产业链垂直整合，新增四板挂牌企业211家。

　　（六）着力抓保障、兜底线，群众生活持续改善。全年民生支出占地方一般公共预算支出比重达到81%，承诺的民生实事全部办结。设立扶贫产业基金2亿元，建设易地扶贫搬迁安置点41个，全年实现75个贫困村“销号”、6.03万贫困人口脱贫。发放创业担保贷款3亿元，扶持创业6200人，带动就业24000人。新建社区居家养老服务中心24个、农村老年人互助照料活动中心64个，建成32所留守儿童服务站、20所青少年“阳光家园”和154所村级“希望家园”。新开工保障性安居工程项目13461套，建成7272套，分配入住5216套。完成42所义务教育学校标准化建设，启动龙泉中学南校、望兵石学校、荆门高新学校、碧桂园学校建设，荆门职业学院对外招生。整合城乡居民医疗保险，综合参保率达到94.6%。启动分级诊疗工作。新建村级文化广场114个、农民体育健身工程100个。成功举办荆门国际马拉松赛、2016荆门爱飞客飞行大会、第十二届中国（荆门）菊花展、第七届亚洲赏鸟博览会、市七运会等。推进基层积分制管理，特殊人群、社会组织、信息网络等服务管理机制不断完善。启动“七五”普法工作。开展“警格对接”试点，实行交巡特警一体化，立体化防控体系不断夯实。强化安全生产责任制，全市未发生重特大安全生产事故。加大矛盾纠纷排查化解力度，社会大局和谐稳定。

　　各位代表，五年风雨兼程，五年砥砺奋进，在荆门发展史册上留下了浓墨重彩的一页。这些成绩的取得，是省委、省政府和市委坚强领导、科学决策的结果，是市人大、市政协和社会各界有效监督、大力支持的结果，是全市上下同心同德、团结奋斗的结果。在此，我代表市人民政府，向全市人民，向人大代表、政协委员，向各民主党派、工商联、人民团体、无党派人士、离退休老同志，向驻荆部队、武警官兵，向关心支持荆门发展的同志们、朋友们，表示衷心的感谢并致以崇高的敬意！

　　回顾过去五年的工作，我们深刻体会到，做好政府工作，必须始终坚持目标导向，勇担历史重任、发展重责,以竞进之姿争得在全省发展格局中的更重份量；必须始终坚持问题导向，加快补齐短板、换道超越，以非常之策突破改革发展的重大瓶颈；必须始终坚持民生导向，着力体察民情、增进民利，以惠民之举提升群众幸福感、获得感；必须始终坚持实干导向，力求抓铁有痕、踏石留印，以务实之风推动各项决策部署落地生根。只要我们一以贯之地秉持这些好经验、好做法、好作风，荆门的改革发展就一定能够增创新优势、迈上新台阶、实现新跨越！

　　在总结成绩的同时，我们也清醒地看到在经济社会发展中还存在一些困难和问题。主要表现为：经济总量不大，产业结构不优，创新能力不足，综合实力有待进一步增强；资源环境制约加剧，大气、水污染问题突出，推进绿色发展任重道远；旧城更新和新区建设任务繁重，交通等重大基础设施建设亟待加快推进；公共产品供给和公共服务水平与群众期盼还有不小差距，群众反映的一些热点难点问题还没有得到有效解决；公务员队伍中不想为、不敢为和不善为的问题仍然存在，等等。对此，我们将高度重视，认真对待，采取更加有力的措施切实加以解决。

　　二、未来五年经济社会发展的总体要求和目标任务

　　未来五年，是我国全面建成小康社会、实现第一个“百年梦想”目标的攻坚决胜期，是湖北构建中部地区崛起重要战略支点、打造长江经济带建设脊梁的黄金发展期，也是我市爬坡过坎、换道超越的战略机遇期。刚刚闭幕的市第八次党代会，提出了奋力实现荆门在湖北中部崛起的战略目标，吹响了荆门新一轮大发展的集结号。

　　未来五年全市经济社会发展的指导思想是：全面贯彻党的十八大、十八届三中、四中、五中、六中全会和习近平总书记系列重要讲话精神，按照统筹推进“五位一体”总体布局、协调推进“四个全面”战略布局的要求，坚持以市第八次党代会精神为指导，以供给侧结构性改革为主线，深入实施中国农谷建设和柴湖振兴发展省级战略，遵循生态立市、产业强市、资本兴市、创新活市的路径，努力建成江汉平原中心城市、湖北新兴增长极、全国新型城镇化和城乡一体化示范区，率先在湖北同类城市全面建成小康社会、基本实现农业现代化、建成国家创新型城市，奋力开启荆门在湖北中部崛起的新征程。

　　各位代表，崛起不仅是总量的扩张、速度的提升，更是发展路径的转变、发展动能的转换、发展维度的拓展，是在新的起点上实现更高质量、更有效益、更加公平、更可持续的发展。实现在湖北中部崛起，是我们必须担当的政治责任、必须把握的历史机遇、必须解答的发展命题、必须回应的群众期盼。我们一定要把思想和行动统一到谋崛起、促崛起、快崛起上来，努力实现以下工作目标：

　　——经济实力更强。主要经济指标增幅高于全省平均水平、领先全省同类城市。人均GDP达到1万美元，地方一般公共预算收入、社会消费品零售总额、固定资产投资分别突破180亿元、1000亿元和3500亿元。

　　——发展质量更高。产业发展迈向中高端水平，战略性新兴产业和高新技术产业增加值占地区生产总值的比重分别达到12%和18%。建成全国新型能源化工基地、全国生态农产品加工基地、全国新能源汽车产业化基地、全国绿色建筑和装配式建筑产业化基地及通用航空新城、健康产业城、国家循环经济示范城。

　　——城乡环境更美。中心城市集聚和辐射能力显著增强，县域城市、中心镇培育成效明显，特色小镇、美丽宜居乡村建设迈上新台阶，常住人口和户籍人口城镇化率分别达到64%、42%。生态环境质量明显改善，空气质量优良天数比例达到85%，地表水断面考核达标率达到100%，森林覆盖率达到35%，节能减排完成省定目标。

　　——群众生活更好。城乡就业更加充分，社会分配更加公平，公共服务和社会保障体系更加完善，居民健康水平达到国家健康城市标准，全面建成小康社会各项指标提前达标。

　　重点抓好以下工作：

　　（一）坚持创新崛起，在转换发展动能上走在全省前列

　　加强科技创新。加快构建以企业为主体、市场为导向、七大主导产业为重点的区域创新体系，打造高新技术产业集聚、科技创新合作、科技创新孵化、科技中介服务等创新驱动平台，引进培育一批高层次创新人才，建成江汉平原科技创新中心、国家现代农业科技创新中心和国家知识产权示范城市。到2021年，新增省级以上工程技术研究中心、工程实验室、企业技术中心、工业设计中心50家，新增院士（专家）工作站20家、产业技术创新联盟10家，高新技术企业达到280家。

　　加强产业创新。着力拓展新空间，释放新需求，创造新供给，大力发展新经济。加快化工、农产品加工、建材等传统产业转型升级，提升通用航空、智能制造装备、再生资源利用与环保等产业实力，培育新能源汽车、电子信息、生物医药、新能源新材料产业发展优势。坚持运用现代发展理念引领农业、现代物质条件装备农业、现代科学技术改造农业、现代经营模式推进农业，构建粮经饲统筹、农牧渔结合、种养加一体、一二三产业融合发展的现代农业产业体系。推动现代物流、总部经济、金融保险、电子商务等生产性服务业向专业化和价值链高端延伸，文化体育旅游、商贸服务、健康养老等生活性服务业向精细化和高品质转变，建成全国知名运动休闲旅游目的地和江汉平原生产性物流基地。加快网络经济发展，促进大数据、云计算和物联网的广泛应用。

　　加强金融创新。大力推进资本兴市，到2021年，社会融资总量达到500亿元，金融业增加值超过100亿元，占地区生产总值比重达到5%。支持已上市挂牌企业定向增发、并购重组，推动更多优质企业上市挂牌融资和发债融资。引进培育金融和类金融机构，建成江汉平原金融保险服务中心。完善“1+N”产业发展基金体系，推行“特色产业链+产业互联网+基金”投资模式，实现七大主导产业链垂直整合。扩大城市发展基金规模，支持城市重大基础设施和新型城镇化建设。大力发展科技金融、绿色金融、普惠金融，加快培育消费金融和财富管理业，催生多种新型金融业态。

　　加强制度创新。以落实“三去一降一补”为重点，深入推进供给侧结构性改革。坚持在行政管理、行政审批、“三农”发展、生态文明建设和社会治理等方面，推出一批标志性、引领性、突破性的改革举措，努力把荆门建设成为湖北全面深化改革的先行区和示范区。完善大众创业万众创新体制机制，扶持发展众创、众包、众扶、众筹，培育一批低成本、便利化、全要素、开放式平台，构建“种子—苗圃—孵化器—加速器—产业园”的梯级孵化体系，努力把荆门建设成为中部地区有重要影响的创新之城、创业之都、创客之乡。

　　（二）坚持协调崛起，在统筹城乡发展上走在全省前列

　　推动区域协调发展。优化城市功能结构和城镇空间布局，打造紧凑之城、宜居之城、智慧之城、书香之城，加快建设现代化精致城市。坚持中心城区“一心三城五组团”发展，不断提升首位度。推动京山县建设华中地区智能产业城，在全省县域经济“第一方阵”争先进位；沙洋县建设汉江流域临港经济示范区，冲刺全省县域经济“第一方阵”；钟祥市建设全国三产融合发展示范县（市），冲刺全国县域经济百强；屈家岭管理区建设国家农业公园；柴湖建设城乡统筹示范区、移民发展样板区、民生改善先行区。

　　推动产城融合发展。全力建设漳河新区，重点发展通用航空、大健康、金融保险、现代商业、文化旅游等产业，打造通用航空新城、绿色生态新区，成为荆门城市新中心。完善掇刀区与高新区一体化发展机制，重点发展化工、高端装备制造、新能源新材料、电子信息、大健康、再生资源利用与环保等产业，创建国家创新型特色园区，冲刺国家高新区“第一方阵”。推动东宝工业园与老城区产城融合，重点发展电子信息、绿色建材、现代物流等产业，建设江汉平原“五化”同步发展先行区，在全省县域经济“第一方阵”争先进位。推动京山温泉新区、沙洋滨江新区、钟祥南湖新区与经济开发区融合发展，实现产业与城市功能融合、空间整合。

　　推动城乡一体发展。推进城乡基础设施互联互通、共建共享，构建城乡公共服务均等化体制机制。以三条新型城镇化示范带为主轴，以小城市、中心镇、新型城镇化试点镇、特色小镇为重点，建设一批经济发达、功能完善、特色鲜明的现代化小城镇。推动农民就地就近转移和农村生产生活方式转变，实现全市三分之一左右的农村人口居住在新型农村社区，三条新型城镇化示范带美丽宜居乡村建设全覆盖。

　　（三）坚持绿色崛起，在生态文明建设上走在全省前列

　　筑牢绿色屏障。树立全域生态理念，落实生态主体功能区规划，加快形成“一带两屏四网六廊”生态安全新格局。严格资源消耗上限、环境质量底线和生态保护红线管控，实行负面清单管理和准入制度管理，加强环境监管网格化管理，推行生态绩效考核，确保“源头严防、过程严管、后果严惩”落到实处。

　　发展绿色产业。大力发展绿色经济，加快传统产业绿色改造，建成以静脉产业园、环保装备产业园、绿色建筑和装配式建筑产业园为重点的绿色产业集群，把荆门绿色生态科技产业城建设成为绿色崛起示范区。强力推进节能减排，促进资源节约高效利用。加快国家循环经济示范市建设，推动荆门化工、荆襄磷化、格林美等循环产业园提挡升级。

　　倡导绿色生活。培育绿色生态文化，构筑绿色人居环境，积极引导绿色消费、节约消费、适度消费，使绿色、低碳、节约成为全社会风尚和全民自觉行动。

　　加强绿色治理。强化生态治理和环境修复，统筹推进治水治气、治土治山、治城治乡，全面实施大气污染治理、水环境综合治理、土壤修复、碳汇林业建设等生态环境治理“十大工程”，让荆门的天更蓝、山更绿、水更清、“颜值”更高、空气更清新！

　　（四）坚持开放崛起，在拓展发展空间上走在全省前列

　　打造对外开放大通道。加快建设“铁、水、公、空、管”多式联运、无缝对接的立体大交通，打造江汉平原区域性综合交通枢纽。全力推进荆荆客专、襄荆宜高铁建设，力争早日迈入“高铁时代”。加强漳河机场、军民合用冷水机场建设，规划建设京山、沙洋、钟祥一类通用机场。加快汉江至长江千吨级骨干航道网建设，建成汉江荆门组合港，打造汉江航运中心。推动公路交通加密、提标，建成“八纵六横一环”的骨干公路网络。

　　建设对外开放大平台。加快临港、临空经济区建设，打造无水港、陆地港，规划建设综合保税物流中心、综合保税区、跨境电子商务和劳务合作等对外开放平台。积极争取设立独立的荆门海关和出入境检验检疫机构，实现区域通关一体化。

　　构建对外开放大方阵。扩大利用外资规模，提高利用外资质量。大力实施外贸出口基地建设工程，培育一批核心竞争力强的外向型企业。对接“一带一路”，鼓励企业“抱团出海”，组建出口型产业联合体，积极开展国际产能合作。

　　（五）坚持共享崛起，在强化民生保障上走在全省前列

　　全力推进精准扶贫。统筹推进“五个一批”工程，深入实施技能培训、创业推进、科技驱动、扶志行动“四大计划”，确保到2018年，实现全市21.93万贫困人口全部脱贫，210个贫困村全部“销号”。

　　全力增加群众收入。坚持以创业带就业、以就业促增收，健全统一开放的人力资源市场和覆盖城乡的公共就业服务体系，统筹推进高校毕业生、农业转移人口、退役军人、城镇就业困难人员稳定就业、充分就业。实施居民收入倍增计划，完善最低工资保障和工资支付保障机制，切实维护劳动者合法权益。不断拓宽财产性收入渠道，稳步增加农民收入。

　　全力加强社会保障。大力实施全民参保计划，基本实现法定人员全覆盖。筑牢以社会救助为重点的兜底保障，确保困难群众基本生活。加快推进住房保障和供应体系建设，在实现住有所居的基础上向住有宜居转变。

　　全力发展社会事业。加快完善现代教育体系，推进学前教育公益普惠发展、义务教育高水平均衡发展、普通高中教育特色化多样化发展、职业教育产教融合式发展、高等教育转型发展。实施健康荆门建设，加强健康管理，改善健康环境，完善医疗保障体系。提高城乡体育设施覆盖率，开展全民健身活动，不断增强人民体质。进一步提升文化软实力，丰富群众精神文化生活。

　　各位代表，崛起的蓝图已经绘就，美好的愿景就在前头。我们坚信，当第一个“百年目标”梦圆之时，一座产业高端、经济繁荣的实力之城，一座城乡协调、特色鲜明的精致之城，一座生态宜居、百姓安康的幸福之城，必将展现在我们面前、崛起在荆楚大地！

　　三、2017年工作安排

　　2017年是实施“十三五”规划的重要一年，是供给侧结构性改革的深化之年，也是新一届政府各项工作的起始之年。做好今年的工作，任务繁重，意义重大。全年主要预期目标是：地区生产总值增长9%左右；规模以上工业增加值增长10.5%；全社会固定资产投资增长19%；社会消费品零售总额增长13%左右；地方一般公共预算收入增长10%；实际利用外资增长8%；出口总额增长9%；城乡常住居民人均可支配收入均增长9%左右；居民消费价格涨幅控制在3.5%以内；城镇登记失业率控制在4.5%以内；人口自然增长率稳定在7‰以内。重点抓好以下工作：

　　（一）深化供给侧结构性改革，加快培育发展新动能

　　切实增加有效供给、减少无效供给，打通供求渠道，进一步提高供给体系的质量和效率。

　　落实“三去一降一补”任务。加大企业兼并重组力度，依法依规处置“僵尸企业”，全年压减低端肥产能50万吨。推进棚改货币化安置，让房地产回归住房属性，确保全市商品住房库存平均去化周期保持在合理区间以内。充分运用股权融资、市场化债转股等多种手段降低杠杆率，力争企业直接融资占全社会融资比重达到30%，新增“新三板”挂牌企业10家、四板挂牌企业200家，发行企业债券40亿元。继续打好降低制度性交易、人工、物流、财务等成本和税费负担“组合拳”，为企业降费让利10亿元以上。坚持既补硬短板也补软短板，既补发展短板也补制度短板，突出产业竞争力、重大基础设施建设、生态环境保护、公共服务等重点领域，精准发力、持续用力，加快突破发展瓶颈。

　　大力发展新经济。以科技创新为支撑，培育壮大战略性新兴产业，力争高新技术产业增加值占地区生产总值比重达到14%，研究与实验发展经费支出占地区生产总值比重达到1.8%。提升园区创新能级，扶持科技型中小企业发展，新增高新技术企业50家，推动沙洋经济开发区晋升为省级高新区。引导企业培育“工匠精神”，与高等院校、科研院所联合组建产业协同创新中心、产业技术创新联盟和制造业创新中心。实施“创新创业在荆门”行动计划，创建省级众创空间2家以上。实施“互联网+云荆门”行动计划，催生更多的新产业、新技术、新业态、新模式。

　　积极推进消费升级。加快中国农谷会展中心、农产品物流园区、临港（临空）物流园区、综合物流园区建设，新增3A级以上物流企业2家。推进核心商圈建设，实施商贸企业成长工程，新增限上企业50家、出口企业10家。加快通用航空文化创意示范区、积分制文化创意产业园等建设。制定全域旅游规划，整合旅游资源，办好品牌节会，实现旅游综合收入165亿元。启动全民健身中心、瑞德康复产业园建设，建立荆门华大基因检验检测中心，推动市中医医院和康复医院建设康复养生护理中心。实施无线城市、光纤入户、“三网”融合和下一代互联网建设工程，加快荆门电子商务综合服务平台建设，建成城市大数据中心。

　　（二）做大做实投资底盘，构筑经济增长新支撑

　　坚定不移地把扩大有效投资作为经济工作的主抓手，为稳增长提供有力支撑，全年固定资产投资突破1800亿元。

　　强力推进产业链招商。完善招商引资“六个一”推进机制，围绕七大主导产业，引进一批产业龙头企业和延链补链强链项目。推行以商招商、委托招商、股权招商、依托基金公司招商等方式，增强招商引资实效。瞄准“一带一路”和珠三角、长三角、东北、京津冀等地区，引进一批优质项目和企业区域性总部。加强招商引资信息研判、项目预审和布局统筹，着力营造法制基础上的良好商业环境，全年实际利用外资突破4亿美元，招商引资实际到位资金突破1000亿元。

　　加快重大项目建设。深化重大项目路线图管理，精准策划项目，抓好项目前期，确保项目落得下、建得好、发展快。在产业项目方面，以146个省市重点项目为引领，统筹推进840个亿元以上项目建设，开工建设金泉新材料二期、通用航空制造业产业园等项目，加快荆门石化千万吨油品质量升级、京山轻机康复机器人、泰富重装沙洋基地、大连数控机床、新能源汽车产业园、万华生态产业园等项目建设，推动亚丹生态家居、宝得瑞健康食品工业园等280个亿元项目建成投产。在交通项目方面，加快推进枣潜高速公路及15个国省道、蒙华铁路荆门段、军民合用冷水机场改扩建等项目建设，确保汉江荆门组合港后港综合码头动工兴建，完成荆荆客专、襄荆宜高铁前期工作，力促开工建设。在水利项目方面，加快汉江以西水系连通及城市备用水源工程建设，实施汉北河流域综合整治和碾盘山水利水电枢纽工程。

　　打通社会资本对接实体经济通道。落实“六个必须”要求，充实重资产建设项目库，完善收益、准入、考核和退出机制，健全租赁回购办法。加快重资产建设进度，开工建设厂房600万平方米以上。开展激活民间投资专项行动，促进民营经济持续健康发展，力争民间投资占全市投资比重达到75%。大力推广PPP模式，全年落地项目不少于30个，融资规模突破150亿元。

　　（三）突出发展先进制造业，再造工业经济新优势

　　全面对接“中国制造2025”，打造工业经济“升级版”，奋力向先进制造业强市迈进。

　　推动传统产业改造升级。以荆门石化、新洋丰、金鹰能源、明弘玻璃等为重点，深入实施“百企技改”行动，完成技改投资320亿元以上。以京山轻机、福耀玻璃、动能体育等为重点，开展百家智能工厂和数字化车间试点示范，加快发展产业链协同制造、个性化定制和电子商务。以埃斯顿、台顺、巨浪等机器人企业为重点，鼓励引导企业实施“机器换人”。以磷化工、建材、农产品初级加工企业为重点，实施兼并重组行动计划，全市兼并重组企业不少于20家。

　　推动新兴产业培育壮大。以通用航空、智能制造装备、新能源汽车、再生资源利用与环保、新一代信息技术、新能源新材料、生物医药等为重点，出台个性化扶持政策，突破性发展战略性新兴产业，确保主营业务收入达到750亿元。依托荆门通用航空产业园、新能源汽车产业园，深化与中航工业、长丰猎豹、亿纬锂能的合作，力争有整机、整车下线。加强园区考核结账，培育20个左右特色产业园区，促进新兴产业集群发展。

　　推动骨干企业做大做强。实施“优质企业倍增工程”和“百名企业家培育工程”，深化“进百企、解难题”活动，打造企业家领军队伍，加快培育一批“专精特新”企业，新增规模企业100家、10亿元企业10家、50亿元企业2家，1家企业入围中国企业或民营企业500强，1家企业入围省100强,新增1个国家级品牌和10个省级品牌。支持京山轻机、中集安瑞科、凯龙化工等企业“走出去”开拓国际市场。

　　（四）加快转变农业发展方式，打造现代农业发展新高地

　　大力发展现代农业，加快农业结构战略性调整，促进农业增效、农民增收。

　　深入推进农业供给侧结构性改革。把增加绿色优质农产品供给放在突出位置，积极推广“香稻嘉鱼”等10种生态种养模式，做大特色产业板块，启动30万亩生态蔬菜基地建设，创建100个农牧结合生态养殖示范场，新增部、省级水产健康养殖示范场8家，新增“三品一标”25个。落实耕地保护共同责任，建立粮食生产功能区、“双低”油菜保护区和果蔬优势生产区。推进高标准农田建设，实施土地整治20万亩，分别在三条新型城镇化示范带沿线连片建设高效生态农业示范区。

　　促进一二三产业融合发展。加快发展现代种业，重点打造马铃薯、甘薯、油菜、龟鳖、湿地松等国家级现代育种基地，推进中国农谷现代种业科技园建设。推动农产品加工向精深化、功能化、品牌化发展，开发富硒、长寿食品，发展休闲、功能食品；鼓励龙头企业打造集种业、基地、加工、销售为一体的全产业链，力争农产品加工业产值增长10%。深化与阿里巴巴集团合作，推进淘宝镇、村建设，实现农村电商服务站（点）全覆盖。加快建设屈家岭农耕文化园、柴湖“中国花城”、彭墩乡村大世界等，大力发展休闲、观光农业。

　　构建农业综合服务体系。健全农村公共服务“以钱养事”机制，依托新型农业经营主体建立农业综合服务超市，开展市场化服务，推行政府购买农业公益性服务。依托京山超能农业装备制造产业园、屈家岭豪丰农机装备产业园，提升农机装备水平。提高荆门农用航空站综合服务能力，推广植保无人机应用。推进农业生产全程机械化，力争主要农作物耕种收综合机械化水平达到78%。加快农业综合信息平台和物联网示范工程建设，提高农业信息化水平。新建、改造泵站300座，整修渠道3000公里，疏浚河道130.5公里，整治塘堰6300口，改善农田灌溉面积30万亩。

　　（五）推进以人为核心的新型城镇化，开创城乡一体化发展新格局

　　按照布局紧凑、品位高端、管理精细、文化浓郁的要求，大力推进生态修复、城市修补，扎实做好国家新型城镇化综合试点工作，力争常住人口城镇化率达到57%、户籍人口城镇化率达到35%。

　　增强中心城区承载力。全年实施城建项目439个，完成投资483.4亿元。倾力建设漳河新区，加快金融保险中心、荆门绿色生态科技产业城核心岛会展中心、通航航管楼、飞行体验中心、爱飞客总部基地等项目建设。改善老城区环境，推进文化宫、七里铺等片区棚户区改造，进一步扩大货币化安置比例；开工建设8公里综合管廊和3平方公里海绵城市，完成16个渍水严重地段整治；实施三条高速公路出入口景观提升工程、东宝山和象山综合整治工程、城市绿地扩建工程，打造城市靓丽风景线。启动罗汉山隧道、东宝山南隧道等控制性节点项目，实施白云大道改造工程，确保荆门城区绕城公路9月底前建成通车。

　　提升城市精细化管理水平。落实《荆门城市管理条例》，巩固和扩大创卫工作成果。持续加强“三无”小区、背街小巷、城乡结合部整治，着力解决群众反映强烈的市容市貌、交通和经营秩序、环境卫生、噪音扰民等问题，构建城市管理工作常态化、长效化机制。坚决打击违建行为，强力拆除存量违建，确保新增违建为零。

　　构建多极多点支撑格局。在三条新型城镇化示范带全域推行PPP模式，抓好彭墩长寿食品产业园、汇源生态产业园、沙洋生态农业风情体验园等一批支撑性项目建设。按照产业“特而强”、形态“精而美”、机制“新而活”的思路，加快培育爱飞客航空小镇、屈家岭中国农谷风情小镇、柴湖花卉小镇、彭墩长寿小镇、孙桥国宝桥米小镇、官垱双低油菜小镇等一批特色小镇。开展农村环境综合整治，完善基础设施，创建20个省级、100个市级美丽宜居示范村。

　　（六）加大生态治理和保护力度，推动环境质量新提升

　　统筹生产生活生态，推进绿色富市、绿色兴业、绿色惠民，厚植生态文明新优势。

　　加强重点领域环境整治。实施“蓝天工程”，打好大气污染防治攻坚战，加强扬尘和挥发性有机物管控，持续做好秸秆禁烧和综合利用工作，加大餐饮油烟污染治理力度，完成中心城区12家采石塘口复绿工程。实施“碧水工程”，加强漳河、温峡、惠亭湖等饮用水源地保护，持续推进竹皮河、天门河、长湖流域治理，全面实施“河长制”“湖长制”，确保城镇污水处理设施全面达到一级A排放标准。实施“净土工程”，抓好中国农谷土壤改良和修复试点项目，加强矿山环境和采空区恢复治理。

　　推进清洁生产和循环经济发展。制定全市节能减排扩容计划，扩大强制性清洁生产企业范围，对13家企业清洁生产进行审核。有序推进电能替代工作。巩固节能减排财政政策综合示范市建设成果，确保通过国家验收。推动工业园区循环式、绿色化改造，加快荆门静脉产业园建设。推进畜禽养殖废弃物处理和资源化，大力发展循环农业。

　　加大生态管控制度建设力度。积极推进环境污染第三方治理，继续实施中心城区空气质量生态补偿制度，完善跨县（市、区）河流断面考核制度，探索实施跨流域生态补偿制度。普遍推行垃圾分类制度，加快建立分类投放、分类收集、分类运输、分类处理的垃圾处理系统。继续开展自然资源资产负债表编制和领导干部自然资源资产审计工作，强化负债表和审计结果运用。严格土地利用总体规划管控，提升节约集约用地水平。完善市、县、镇、村四级联创机制，加快推进国家生态文明建设示范市、国家环境保护模范城市创建。

　　（七）创新体制机制，实现重点领域改革新突破

　　着力推进重大改革措施落地见效，为经济社会发展增活力、添动力。

　　扎实推进“放管服”改革。推行“互联网+”政务服务，加强行政审批信息化、标准化建设，再减事项、再优流程，完善“一窗受理、一章审批、一站服务、一次评估、一网运行、一门通办”运行机制。培育规范中介服务市场，建好用活中介服务超市。完善和落实监管清单制度，全面推行“双随机一公开”监管改革。加强基层便民服务体系建设，编制公共服务事项目录和办事指南，升级政务服务、公共资源交易、公共检验检测等平台。整合执法资源，深入推进农业、商务、文化、城管等综合执法体制改革。分类推进国有企业改革，完成市直机关所属企业及经济实体脱钩改制。

　　大力推进财税和投融资体制机制改革。深化预算管理制度改革，推进财政专项资金统筹使用。加强税收共治，创新财源建设机制。完善政银企深度对接机制，推行主办银行制度，充分发挥政银合作基金杠杆作用，新增社会融资200亿元以上，表内贷款余额超过1000亿元。深化投融资平台改革，确保规范高效运营。做大母基金，优化子基金，推动各类基金实质性运转。推广金融服务网格化和金融服务中心模式。强化信用体系建设，积极防范和处置金融风险。

　　积极推进农村综合改革。推广“沙洋模式”，确保按户连片耕种率达到75%。鼓励多种形式的农村土地流转和适度规模经营，新建土地股份合作社100家。推广京山农村集体资产股份权能改革试点经验，通过股份合作、直接租赁、市场运作，增加农民财产性收入。推广钟祥农村承包土地经营权抵押贷款试点经验，激活农村各类生产要素。着力盘活“三资”，发展壮大村级集体经济。扎实推进供销社、国有林场和农业水价综合改革。

　　（八）坚持共建共享，满足群众生活新期盼

　　大力实施改善民生行动计划，坚守底线、突出重点、完善制度、引导预期，让改革发展成果更多惠及人民群众。

　　打好脱贫攻坚战。按照“六个精准”和“精准扶贫、不落一人”要求，加快发展脱贫产业，大力实施健康扶贫，推进农村低保与扶贫开发有效衔接，投放扶贫小额信贷2亿元以上，实现77个贫困村“销号”、6.17万名贫困人口脱贫。强化脱贫攻坚责任，完善考核、评估、退出机制。

　　切实加强民生保障。开展“创业+”系列培训，发放创业担保贷款2亿元以上，实现困难人员再就业4000人、下岗失业人员再就业1万人。全面实施养老保险制度改革。开展长期护理保险全国试点工作。鼓励发展居家养老和社区养老，提高养老服务质量。完善农业转移人口住房公积金制度，扩大住房公积金制度覆盖面。提高最低生活保障标准和生活补助水平，强化城乡困难群体基本生活兜底保障。

　　协调发展社会事业。坚持教育优先发展，创建省学前教育示范区，支持荆楚理工学院转型为应用型大学，加快推进通用航空学院、健康护理学院、积分制商学院等一批特色职业学院建设，促进信息技术与各类教育深度融合。扎实推进医改和分级诊疗工作，构建市、县、乡（社区）一体的紧密型医联体，启动荆门市疾控中心建设。落实全面两孩政策，实施妇幼健康筛查免费项目，推进基本生育免费服务全覆盖。鼓励文艺精品创作，繁荣和发展哲学社会科学，推进传媒融合发展，办好首届漳河国际冬泳节、全国U系列足球联赛和市第九届老年人体育健身大会。扎实开展第三次农业普查。

　　提高社会治理水平。抓好法制宣传教育和公民道德建设，健全公共法律服务体系。加强和创新社会治安综合治理，深化警务体制机制改革，构建动态化、信息化、一体化治安防控体系，建设更高水平的“平安荆门”。完善应急管理体制机制，提高防范和处置各类突发事件的能力。落实安全生产责任制，实施“两个全覆盖”工程，坚决遏制各类重特大安全事故发生。强化食品药品监管，健全农产品质量追溯体系。加强村（社区）群众自治组织建设，提升社区标准化建设水平，大力培育社会组织。依法管理宗教事务，落实民族政策，推进对外援建工作。充分发挥工会、共青团、妇联、科协、侨联、工商联、残联等群团组织在服务经济社会发展中的重要作用。进一步抓好保密、对台、档案、地方志、物价、移民、地震、气象、人防、老龄、机关事务、外事侨务等各项工作。加强国防动员和后备力量建设，推进军民融合发展。

　　各位代表，在做好以上各项工作的同时，我们还将着力办好以下民生实事：

　　1．完成易地扶贫搬迁2000户。新开工保障性安居工程19938套，基本建成8926套，分配入住4000套。

　　2．新增城镇就业4.5万人，组织就业培训1.6万人,促进5000人创新创业。

　　3．完成39所义务教育学校标准化改造。新建、改扩建幼儿园25所，乡镇及以下入园幼儿享受普惠性服务率达到90%。

　　4．推进中心城区公共工程建设，建成龙泉中学南校、荆门剧院、南北汽车客运站，完成市博物馆、市民中心、传媒中心、社会福利中心、“两馆两中心”主体工程建设，开工建设市科技馆。新增公共停车泊位2000个。开工建设11个小游园，完成南片区公园、双泉公园、海慧小游园、缸盖厂小游园、关坡小游园、月亮湖小游园建设。

　　5．关停天门河流域禁养区内养殖场，拆除长湖养殖围网、漳河水库筑坝拦汊，在100个村建成农村生活污水生态化、微动力或无动力处理设施。关停或改造215台20蒸吨以下燃煤锅炉，淘汰所有黄标车，实施电厂超低排放改造。

　　6．完成500公里农村断头路及水毁路改造升级。新建或改造1125个农村电网配电台区。解决10万农村居民饮水安全问题。

　　7．建成农村幸福院暨老年人互助照料活动中心40个、城市社区居家养老服务中心11个。

　　8．完成182个“五化”村卫生室、6个“四化”乡镇卫生院创建工作，达标率实现100%。

　　9．新增全民健身路径工程100套、村级农民体育健身工程100个。建设县级图书馆、博物馆、文化馆，提挡升级乡镇（街道）中心文化站13个。

　　10．拓展网格化社会管理平台功能，在所有城镇社区和136个农村试点社区推行积分制管理。新建中心城区8个警务综合服务站和4个进出城区治安卡口。

　　各位代表！民生无小事，枝叶总关情。这些实事无论任务有多重、工作有多难、资金有多紧，我们都务求保质保量、一件不差、一丝不苟地落实好，努力让全面小康的梦想照进生活、成为现实，让发展更有“温度”、幸福更有“质感”，让全市人民感受到每年都有新变化、一年更比一年好！

　　四、加强和改进政府自身建设

　　站在发展新起点，开启崛起新征程，政府必须有新作风、新作为、新形象。我们将始终坚持“发展为先、民生为本、担当为要”，切实增强干事动力、办事能力、成事效力，努力建设敢为、善为、有为的公务员队伍，着力打造法治政府、服务型政府和廉洁政府。

　　（一）强力治“散”，重规矩、严法治。坚持把政治纪律和政治规矩挺在前面，切实增强“四个意识”，确保令行禁止、政令畅通。坚持把团结协作、和衷共济贯穿始终，自觉接受市委集中统一领导，主动接受市人大及其常委会的法律监督、工作监督和市政协的民主监督，维护来之不易的大好发展局面。坚持把法治精神融入血脉，加强政府法治能力建设，推进机构、职能、权限、程序、责任法定化，做到法定职责必须为、法无授权不可为，把政府工作全面纳入法治轨道。

　　（二）强力治“懒”，重效能、抓落实。坚持简政不减责、放权不放任，构建雷厉风行的落实链条，做到事有专管之人、人有明确之责、责有限定之期。坚持一环接着一环拧、一锤接着一锤敲，积小胜为大胜，积跬步至千里。推行岗位责任制、首问负责制、一线办公制、服务承诺制、限时办结制，着力解决懒政、怠政、庸政等问题，确保人人高效率、事事快节奏、处处见实效。

　　（三）强力治“软”，重担当、争一流。大力倡导和弘扬担当文化，深入项目建设、园区建设第一线，深入信访维稳、深化改革最前沿，积极化解矛盾，着力解决问题。坚持在政策之内抢着干、政策之外探索干，以敢闯敢试的魄力创新求变。引导全市公务员敢于拉高标杆，干一流工作，创一流业绩，树一流形象。严肃行政问责，真正做到不尽责就追责、不作为就让位、不发展就受罚。完善容错纠错机制，真正为担当者担当，给实干者实惠。

　　（四）强力治“浮”，重服务、暖民心。坚持下沉到群众中间，多走乡间路、多进企业门、多听百姓声。带着感情、带着责任、带着敬畏，多干有质量、有效益的事，多干打基础、利长远的事，多干顺民意、惠民生的事，努力让基层满意、群众满意、市场主体满意，让老百姓的荷包更鼓、心情更好、笑容更多、幸福感更强。

　　（五）强力治“奢”，重廉洁、扬正气。压实全面从严治党主体责任，严格遵守中央八项规定精神、省委六条意见和市委实施细则，做到心有所畏、言有所戒、行有所止。切实抓好公共资金、公共资源、国有资产监管，加强行政监察、财政和审计监督，推行重大决策和制度廉洁性评估，把行政权力落在清单上、晒在阳光下、关在笼子里。坚决纠正损害群众利益的不正之风，对各类违法违纪案件“零容忍”、严查处，让全市公务员队伍不敢腐、不能腐、不想腐！

　　各位代表，我们正处在改革与创新的伟大时代，处在转型与竞进的黄金时期，奋力崛起的号角奏响，换道超越的步伐铿锵。让我们紧密团结在以习近平同志为核心的党中央周围，在省委、省政府和市委的坚强领导下，坚定发展信心、凝聚发展合力、升腾发展气场，为荆门加快实现“三大定位、三个率先”、在湖北中部崛起而努力奋斗！
